# Knocking down of LINC01220 inhibits proliferation and induces apoptosis of endometrial carcinoma through silencing MAPK11

**DOI:** 10.1042/BSR20181794

**Published:** 2019-07-26

**Authors:** Yong Li, Chengcai Kong, Chaoying Wu, Yingqiao Wang, Boqun Xu, Shenglian Liang, Xiaoyan Ying

**Affiliations:** 1 Department of Gynecology, The Second Affiliated Hospital of Nanjing Medical University, Nanjing, Jiangsu 210000, China; 2 Department of Gynecology, The Affiliated Changzhou Maternity and Child Health Care Hospital of Nanjing Medical University, Changzhou, Jiangsu 213000, China

**Keywords:** Apoptosis, endometrial carcinoma, LINC01220, MAPK11, Proliferation

## Abstract

**Background:** Endometrial carcinoma (EC) still threatens the health of women. Thus, to explore how long intergenic non-protein coding RNA 01220 regulates the development of EC.

**Methods:** Whole genome expression profile data of EC and paracancerous tissues in TCGA database were downloaded. LINC01220 expression in EC and paracancerous tissues of patients in our hospital were detected by qRT-PCR. Furthermore, the relationship between LINC01220 expression and clinicopathological features of EC patients was analyzed. After transfection with sh-LINC01220 and pcDNA-MAPK11 (mitogen-activated protein kinase) in EC cells, proliferative, colony formation abilities and apoptosis were determined by cell counting kit-8 (CCK-8), colony formation assay and flow cytometry, respectively. Western blot was conducted to determine the regulatory role of LINC01220 on MAPK11.

**Results:** TCGA data showed that LINC01220 expression is markedly higher in EC tissues than that of paracancerous tissues, which was consistent without detection in EC patients of our hospital. LINC01220 expression was positively correlated to pathological grade and International Federation of Gynecology and Obstetrics (FIGO) stage of EC patients. After knockdown of LINC01220 in EC cells, proliferative and colony formation abilities decreased, whereas apoptotic rate increased. Cor function analysis revealed the positive correlation between LINC01220 and MAPK11 in EC. MAPK11 expression was regulated by LINC01220 in EC cells. Overexpression of MAPK11 can reverse the tumor suppressing effect of LINC01220 on EC.

**Conclusions:** LINC01220 promotes EC development by stimulating proliferation and inhibiting apoptosis of EC cells through up-regulating MAPK11.

## Background

Endometrial carcinoma (EC) belongs to malignant tumor occurring in the endometrium, and most of EC originates in the endometrial glands. EC is common in the female reproductive tract, accounting for 20–30% of female germ cell tumors [1]. In developed countries, EC ranks fourth in female malignant tumors, second only to breast cancer, colorectal cancer and lung cancer. It ranks seventh in female tumors in developing countries [2]. Current treatments of EC mainly include hormone therapy, hysterectomy and combination therapy of chemotherapy and radiotherapy. These measures are effective for early-stage EC. However, advanced, poorly differentiated or special types of EC are difficult to be treated and may ultimately produce poor results [3,4]. Most EC patients experience tumor recurrence or metastasis [5]. Therefore, exploring the molecular mechanisms, identifying new diagnostic and prognostic markers, and exploring new therapeutic strategies for EC are critical to improve the clinical outcomes.

Long noncoding RNA (lncRNA) is a kind of RNA molecule in eukaryotes with a transcript of over 200 nt in length, and it could not encode protein. LncRNA could regulate gene expressions at epigenetic, transcriptional and post-transcriptional levels [6–8]. LncRNAs are divided into five types according to the positions, including sense lncRNA (sense lncRNA), antisense lncRNA, bidirectional lncRNA (bidirectional lncRNA), intronic lncRNA (intronic lncRNA) and larger intergenic noncoding RNA (lincRNA). The ratio of lncRNAs in all RNAs is far more higher than those of mRNAs and miRNAs, which accounts for over 90%, whereas the ratio of mRNAs is only approximately 2% [7,9]. Accumulating studies have shown that lncRNA exerts an important role in tumor development, which undoubtedly brings new hopes in tumor treatment [10–12]. Clarification of the specific mechanism of lncRNAs in EC development contributes to improve EC treatment, which is of great significance in enhancing survival rate of EC patients.

Mitogen-activated protein kinases (MAPKs) are a group of protein kinases containing a threonine/tyrosine residue with a molecular weight of 40–46 kDa [13]. MAPKs are regulated by stimuli, such as extracellular growth factors and differentiation factors, and exert a key role in cell proliferation and differentiation. In the absence of stimulation, intracellular MAPK is dephosphorylated. MAPK is activated only when its threonine and tyrosine residues are phosphorylated. Meanwhile, MAPK is a common pathway or junction for the transmission of intracellular information about the transcription and expressions of genes to the nucleus. MAPK consists of four subunits, namely MAPK14, MAPK11, MAPK12 and MAPK13, of which MAPK14 and MAPK11 are the most important types. Studies have shown that MAPKs are greatly involved in the occurrence and development of various tumors. MAPK11 is highly expressed in breast cancer cells, and is capable of enhancing osteoclast formation and bone resorption [14]. Inhibition of MAPK14 is effective in reducing metastasis of breast cancer cells [15,16]. In glioblastoma, apoptosis can be inhibited by the MAPK and p53 pathways [17]. MAPK can be used as a potential target for the treatment of hematological malignancies [18]. However, the mechanism of MAPK in EC is rarely explored.

## Methods

### Sample collection

Thirty EC patients treated in The Second Affiliated Hospital of Nanjing Medical University from the years 2014 to 2018 were enrolled and they did not receive any preoperative treatments. All harvested EC tissues were pathologically diagnosed. Paracancerous tissues were harvested 2 cm away from the distal end of the EC tissue and confirmed without any infiltration of tumor tissues. Samples were preserved in liquid nitrogen for extracting RNA and proteins. Sample collection was obtained from the written informed consent of patients and approved by the Ethics Committee of Nanjing Medical University.

### Data acquisition

LncRNA expression data of EC were downloaded from the TCGA database (https://tcga-data.nih.gov/tcga/) using the Bioconductor/TCGA biolinks function package. Differentially expressed lncRNAs were analyzed using the edger package.

### Gene set enrichment analysis

Analysis was performed using GSEA 2.2.1 software. Dataset of c2.cp.kegg.v5.1.symbols.gmt was obtained from the MsigDB database in Gene set enrichment analysis (GSEA) website and the enrichment analysis was conducted using the default weighted enrichment statistics method. The random combination number was set as 1000 times.

### Cell culture and transfection

Three EC cell lines (KLE, Ishikawa and RL95-2) were obtained from ATCC. Cells were cultured in DMEM containing 10% FBS (fetal bovine serum), 100 U/ml penicillin and 100 μg/ml streptomycin (HyClone, South Logan, UT, U.S.A.). Cells were incubated in a 5% CO_2_ incubator at 37°C.

For cell transfection, 1.5 ml of serum-free DMEM and 500 μl mixture containing transfection reagent and Lipofectamine™ 2000 were added in each well. After 4–6 h, complete medium was replaced. Plasmids of sh-LINC01220, shRNA-NC, pcDNA-MAPK11 and pcDNA-NC were constructed by GenePharma (Shanghai, China). Plasmid sequence was: sh-LINC01220: 5′-GAGGCCUAUAAUGCAAAGATT-3′.

### qRT-PCR

Total RNA in 30 mg EC tissues or 2 × 10^5^ EC cells was extracted using TRIzol method for reverse transcription according to the instructions of PrimeScript RT reagent Kit (Takara, Tokyo, Japan). RNA concentration was detected using spectrometer. qRT-PCR was then performed based on the instructions of SYBR Premix ExTaq™ (Takara, Tokyo, Japan). The relative gene expression was calculated using 2^−Δ*C*^_t_ method. Primers used in the study were as follows:

GAPDH (forward): 5′-CGGAGTCAACGGATTTGGTCGTAT-3′;

GAPDH (reverse): 5′-AGCCTTCTCCATGGTGGTGAAGAC-3′;

LINC01220 (forward): 5′-CCTTCCATGCTGAGCTGCT-3′;

LINC01220 (reverse): 5′-TGTTGGTGGGATCCAGGAAA-3′;

MAPK11 (forward): 5′-GCTGTCTCGCCCTTTCCAATC-3′;

MAPK11 (reverse): 5′-CGTGCTTCAGGTGCTTGAGTAG-3′.

### Western blot

EC cells were lysed to harvest total cellular protein, followed by determination of total protein concentration. An equal amount of protein sample was loaded on to a 10% SDS/PAGE and then transferred to a polyvinylidene fluoride (PVDF) membrane. Membranes were blocked with skim milk, and incubated with primary antibody (Cell Signaling Technology, Danvers, MA, U.S.A.) overnight at 4°C. On other day, membranes were incubated with horseradish peroxidase (HRP)–conjugated secondary antibody for 2–3 h at room temperature. Finally, an image of the protein band was captured by the Tanon detection system using ECL reagent (Thermo, Waltham, MA, U.S.A.).

### Cell counting kit-8

Transfected EC cells for 24 h were seeded into 96-well plates with 2 × 10^5^ per well. Ten microliters of cell counting kit-8 solution (CCK-8, Dojindo, Kumamoto, Japan) was added in each well after cell culture for 2 days. The absorbance at 450 nm of each sample was measured by a microplate reader (Bio-Rad, Hercules, CA, U.S.A.).

### Apoptotic determination

EC cells were seeded in the six-well plates and transfected for 72 h. Cells were incubated with 5 ml of pre-cooled 70% ethanol overnight at 4°C. On other day, 3 μl of RNase-A was added until the dose of 50 μg/ml for digestion in warm water bath for 30 min. Propidium iodide was then added until the dose of 100 μg/ml and incubated in ice without light for 30 min. Apoptotic rate was determined using flow cytometry.

### Colony formation assay

EC cells in the logarithmic growth phase were seeded in the culture dish with 1000 cells per dish, respectively. After cell culture for 2–3 weeks until visible colony formation, cells were washed with PBS and fixed with 4% paraformaldehyde. Colonies were stained with Crystal Violet for 10–30 min and captured using the microscope.

### Statistical analyses

SPSS 22.0 statistical software was used for data analysis. Data were expressed as mean ± standard deviation (x ® ± s). Differences between the two groups were compared with the *t* test. Cor function was used for comparison of gene correlations. A gene set with a false discovery rate (FDR) < 0.25 was utilized as a significantly enriched gene set in the GSEA. *P*<0.05 considered the difference was statistically significant.

## Results

### High expression of LINC01220 in EC

**Figure 1:**
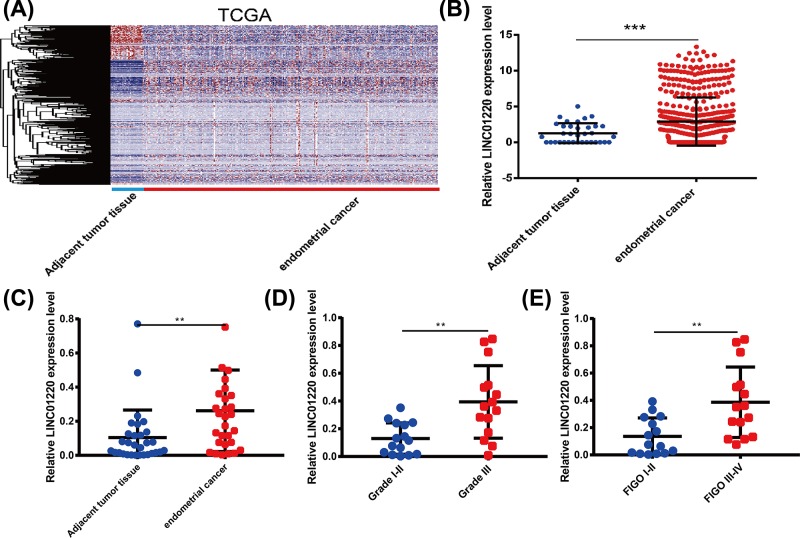
High expression of LINC01220 in EC (**A**) Heat map of differentially expressed lncRNAs in EC of TCGA database. (**B**) TCGA data showed that LINC01220 expression is markedly higher in EC tissues than that of paracancerous tissues. (**C**) LINC01220 expression was higher in EC tissues than that of paracancerous tissues of 30 EC patients enrolled at our hospital. (**D**) EC patients with Grade III had higher expression of LINC01220 than those with Grade I–II. (**E**) EC patients with FIGO III–IV showed higher expression of LINC01220 compared with those with FIGO I–II. **P<0.01, ***P<0.001.

To explore the role of lncRNAs in EC, we first downloaded the whole genome expression profile data of EC and paracancerous tissues in TCGA database (Figure 1A). Among them, LINC01220 expression was markedly higher in EC tissues than that of paracancerous tissues, and the differential expression ratio was the highest (Figure 1B). Next, qRT-PCR was performed to detect the expression of LINC01220 in EC and paracancerous tissues of patients in our hospital. The results also showed the higher expression of LINC01220 in EC tissues than that of paracancerous tissues in 30 EC patients enrolled in our hospital (Figure 1C). Furthermore, the relationship between LINC01220 expression and clinicopathological features of EC patients was analyzed. As the data indicated, LINC01220 expression was associated with International Federation of Gynecology and Obstetrics (FIGO) of EC patients. EC patients with Grade III had higher expression of LINC01220 than those with Grades I–II (Figure 1D). Besides, EC patients with FIGO III–IV showed higher expression of LINC01220 compared with those with FIGO I–II (Figure 1E). These data suggested that LINC01220 may be closely related to the development of EC.

### Knocking down of LINC01220 inhibited proliferation and induced apoptosis of EC cells

**Figure 2:**
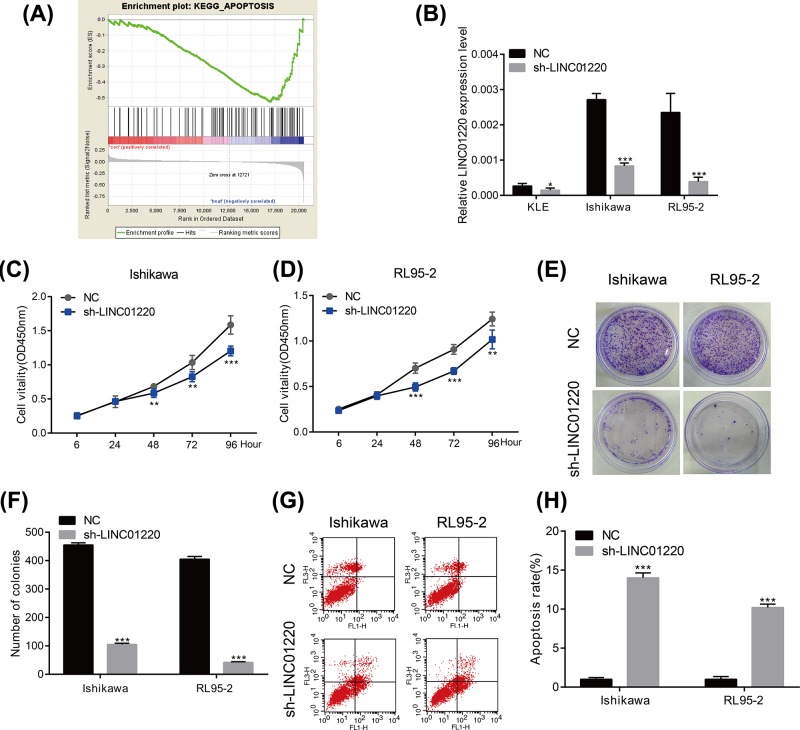
LINC01220 promoted proliferation and inhibited apoptosis of EC cells (**A**) GSEA results showed that the main function of LINC01220 is enriched in apoptosis. (**B**) Transfection efficacy of sh-LINC01220 in KLE, Ishikawa and RL95-2 cells. (**C**) OD value of Ishikawa cells transfected with sh-LINC01220 gradually decreased after cell culture for 6, 24, 48, 72 and 96 h, respectively. (**D**) OD value of RL95-2 cells transfected with sh-LINC01220 gradually decreased after cell culture for 6, 24, 48, 72 and 96 h, respectively. (**E,F**) Colony formation abilities of Ishikawa and RL95-2 cells reduced after LINC01220 knockdown. (**G,H**) Apoptotic rate of Ishikawa and RL95-2 cells remarkably increased after LINC01220 knockdown. *P<0.05, **P<0.01, ***P<0.001.

To further explore the biological function of LINC01220, GSEA was conducted. The results showed that the main function of LINC01220 is enriched in apoptosis (Figure 2A). Subsequently, sh-LINC01220 was constructed and verified for its transfection efficacy in three EC cell lines. Among them, LINC01220 expression markedly decreased in Ishikawa and RL95-2 cells, which were selected for the following experiments (Figure 2B). CCK-8 results revealed that OD value of EC cells transfected with sh-LINC01220 gradually decreased after cell culture for 6, 24, 48, 72 and 96 h, respectively (Figure 2C,D). Similarly, colony formation abilities of Ishikawa and RL95-2 cells reduced after LINC01220 knockdown (Figure 2E,F). Moreover, apoptotic rate of EC cells remarkably increased after LINC01220 knockdown (Figure 2G,H). These results indicated that knockdown of LINC01220 can inhibit the proliferation and clonality of EC cells and promote apoptosis *in vitro*.

### MAPK11 promoted EC development and is regulated by LINC01220

**Figure 3:**
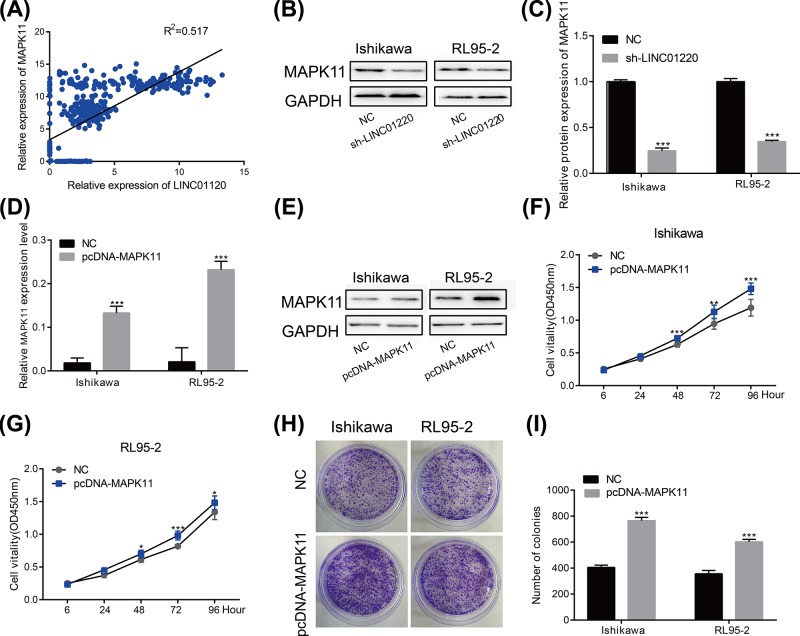
MAPK11 promoted EC development (**A**) Cor function results showed that LINC01220 expression is positively correlated with MAPK11. (**B,C**) LINC01220 knockdown down-regulated protein level of MAPK11 in Ishikawa and RL95-2 cells. (**D,E**) LINC01220 knockdown down-regulated mRNA level of MAPK11 in Ishikawa and RL95-2 cells. (**F,G**) MAPK11 overexpression enhanced proliferative rate of Ishikawa and RL95-2 cells. (**H,I**) MAPK11 overexpression enhanced colony formation ability of Ishikawa and RL95-2 cells. *P<0.05, **P<0.01, ***P<0.001.

We evaluated the correlation between LINC01220 expression and the genome-wide gene in EC expression profiles by the Cor package. The results showed that LINC01220 expression is positively correlated with MAPK11 (Figure 3A). A large number of studies have reported that MAPK11 serves as an oncogene in tumors [19]. In this study, LINC01220 knockdown down-regulated protein level of MAPK11 in Ishikawa and RL95-2 cells (Figure 3B,C). Then the pcDNA-MAPK11 was transfected to Ishikawa and RL95-2 cell lines and the results showed that the mRNA and protein levels of MAPK11 were significantly up-regulated (Figure 3D,E). Furthermore, MAPK11 overexpression enhanced proliferative rate of EC cells (Figure 3F,G), as well as their colony formation ability (Figure 3H,I). The above results indicated that MAPK11 promotes the progression of EC and is regulated by LINC01220.

### MAPK11 overexpression reversed the knockdown effect of LINC01220 on EC cells

**Figure 4:**
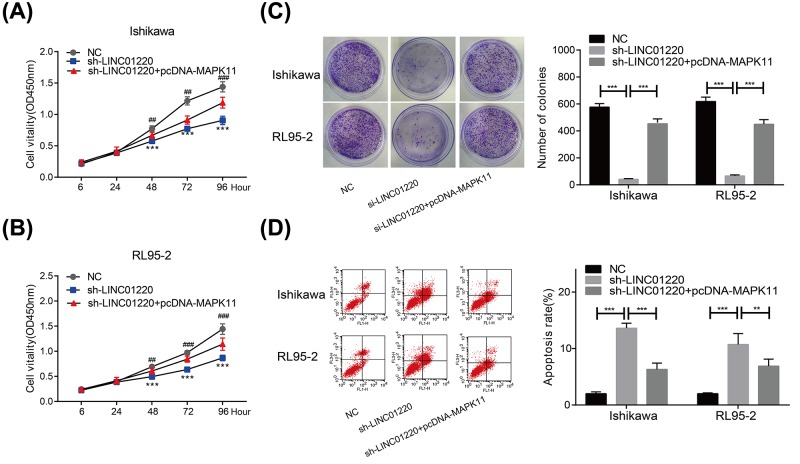
MAPK11 overexpression reversed the effect of LINC01220 on EC cells (**A,B**) LINC01220 knockdown reduced proliferative ability of Ishikawa and RL95-2 cells, which was enhanced after co-transfection with sh-LINC01220 and pcDNA-MAPK11. (**C**) LINC01220 knockdown reduced colony formation ability of Ishikawa and RL95-2 cells, which was enhanced after co-transfection with sh-LINC01220 and pcDNA-MAPK11. (**D**) LINC01220 knockdown induced apoptosis of Ishikawa and RL95-2 cells, which was inhibited after co-transfection with sh-LINC01220 and pcDNA-MAPK11. *P<0.05, **P<0.01, ***P<0.001, ^#^*P*<0.01 (*vs*. sh-LINC01220), ^#^*P*<0.001 (*vs*. sh-LINC01220).

To further examine the roles of MAPK11 and LINC01220 in EC, we knocked down LINC01220 and overexpressed MAPK11 in EC cells at the same time. LINC01220 knockdown reduced proliferative ability of Ishikawa cells, which was enhanced after MAPK11 overexpression (Figure 4A). Similar results were obtained in RL95-2 cells as well (Figure 4B). Subsequently, colony formation ability was determined in co-transfected EC cells. LINC01220 knockdown markedly decreased colony formation ability of EC cells, whereas co-transfected cells showed an enhanced ability (Figure 4C). Rescue experiments were also conducted to determine cell apoptosis in co-transfected EC cells. Previous experiments have already found the stimulated cell apoptosis after LINC01220 knockdown. However, apoptotic rate of EC cells markedly decreased after co-transfection with sh-LINC01220 and pcDNA-MAPK11 (Figure 4D).

The above results suggested that MAPK11 can reverse the tumor suppressing effect of LINC01220 on EC.

## Discussion

EC is a malignant tumor of the female reproductive system. The incidence of EC has increased year by year, which has seriously threatened women’s physical and mental health. It is found that approximately 20% of EC patients have a clear family history. Besides, long-term sustained estrogen stimulation, obesity, infertility, polycystic ovary syndrome and other factors are related to the occurrence of EC. Similar to other malignant tumors, EC results from a slow and complex process involving oncogene activation and tumor-suppressor gene inhibition. Therefore, it is imperative to explore the pathogenesis of EC, so as to develop new targeted therapy.

LncRNA was originally thought to be a ‘transcriptional noise’. As it has been discovered to be dysregulated in a variety of tumor tissues, and involved in apoptosis, invasion and metastasis of tumor tissues, lncRNAs are expected to become new targets for tumor diagnosis and treatment [20]. According to the different roles of lncRNAs in tumor development, they are divided into oncogenes and tumor-suppressor genes. LncRNAs as oncogenes are usually up-regulated in tumor tissues that promote tumorigenesis. For example, lncRNA MALAT1 is highly expressed in prostate cancer, which promotes the development of prostate cancer by activating EZH2 [21]. LncRNA H19 is highly expressed in colorectal cancer cells, and can be utilized as miRNA sponges to promote the transformation of epithelial cells into stromal cells, as well as promote the proliferation and carcinogenic activities of colorectal cancer cells [22]. LncRNA-UCA1 is highly expressed in non-small cell lung cancer cells and promotes its development by targeting miR-193a-3p [23]. The second type of lncRNAs are tumor-suppressor genes, which are usually down-regulated in tumor tissues and inhibit tumorigenesis and development. For example, lncRNA NBAT-1 is lowly expressed in ovarian cancer tissues, and inhibits proliferation, migration and invasion of ovarian cancer cells [23]. LncRNA MEG3 is lowly expressed in breast cancer cells, which is capable of inhibiting proliferation, migration and transformation of epithelial cells into stromal cells by targeting miR-412 [24].

Relative studies have shown that lncRNA ABHD11-AS1 is highly expressed in EC tissues, and promotes proliferation, invasion, migration and progression into G_1_/S phase by up-regulating cyclin D1, CDK1, CKD2, CDK4, vEGF and other factors of EC cells [25]. LncRNA TDRG1, MEG3 and MIR22HG have been found to exert important roles in EC [26–28]. LINC01220 is located on chromosome 14q24.3 and has not been reported in tumor diseases yet. In the present paper, TCGA data analyses found that LINC01220 is highly expressed in EC tissues than paracancerous tissues, which was consistent with the detection in EC patients of our hospital. Besides, LINC01220 expression was positively correlated with pathological grade and FIGO staging of EC patients. *In vitro* experiments indicated that LINC01220 knockdown decreased proliferative and colony formation abilities, but induced apoptosis of EC cells. These results strongly suggested that LINC01220 is expected to be a new target for the diagnosis of EC. MAPKs are a family of conserved serine/threonine protein kinases that are involved in basic cellular development processes, such as proliferation, differentiation, stress response, apoptosis and survival [29]. Abnormal activation of the MAPK pathway has been found in a variety of human tumors, indicating its vital role in tumorigenesis. MAPK activation promotes cancer progression and resistance to chemotherapeutic drugs [30,31]. On the contrary, inhibition of MAPK pathway inhibits tumor metastasis and tumor-related angiogenesis [32,33]. However, studies of MAPK pathway in EC have rarely been reported. Cor function analysis conducted in the present study showed that LINC01220 is positively correlated with MAPK11 in expression profiles of EC. Ater LINC01220 knockdown in EC cells, both protein and mRNA levels of MAPK11 markedly decreased. Overexpression of MAPK11 in EC cells elevated proliferative and colony formation abilities. More importantly, MAPK11 can reverse the effect of LINC01220 on endometrial cancer cells. These results suggested that LINC01220 can promote the progression of EC by regulating MAPK11.

However, the present study still had certain deficiencies. First of all, this research was mainly carried out by *in vitro* cell model. *In vivo* functional researches could further improve the credibility of our results. Second, the way that LINC01220 regulated MAPK11 in EC cells has not been fully explained. In the future experiments, cell localization and potential binding protein of LINC01220 are needed to be explored.

In summary, LINC01220 promotes EC development by stimulating proliferation and inhibiting apoptosis of EC cells through up-regulating MAPK11.

## Abbreviations

ABHD11-AS1: ABHD11 antisense RNA 1
ATCC: American type culture collection
CCK-8: cell counting kit-8
CDK: cyclin dependent kinase
EC: endometrial carcinoma
EZH2: enhancer of zeste 2 polycomb repressive complex 2 subunit
FIGO: International Federation of Gynecology and Obstetrics
GAPDH: glyceraldehyde-3-phosphate dehydrogenase
GSEA: gene set enrichment analysis
LINC01220: long intergenic non-protein coding RNA 1220
lncRNA: long noncoding RNA
MAPK: mitogen-activated protein kinase
MEG3: maternally expressed 3
MIR22HG: microRNA-22 host gene
NBAT1: neuroblastoma associated transcript 1
OD: optical density
qRT-PCR: quantitative real time polymerase chain reaction
TCGA: the cancer genome atlas
TDRG1: testis development related 1
vEGF: vascular endothelial growth factor
